# Prison mental health in South‐East Asia: A narrative review

**DOI:** 10.1002/brb3.70004

**Published:** 2024-08-25

**Authors:** S. M. Yasir Arafat, Sujita Kumar Kar, Chittahari Abhayanayake, Pawan Sharma, M. Marthoenis

**Affiliations:** ^1^ Department of Psychiatry Bangladesh Specialized Hospital Dhaka Bangladesh; ^2^ Biomedical Research Foundation Dhaka Bangladesh; ^3^ Department of Psychiatry King George's Medical University Lucknow Uttar Pradesh India; ^4^ Department of Forensic Psychiatry National Hospital Kandy Kandy Sri Lanka; ^5^ Department of Psychiatry School of Medicine Patan Academy of Health Sciences Lalitpur Nepal; ^6^ Department of Psychiatry and Mental Health Nursing Universitas Syiah Kuala Banda Aceh Indonesia

**Keywords:** forensic psychiatry, incarceration, mental health, prison psychiatry, South Asia

## Abstract

**Background:**

South‐East Asia is a densely populated region with a considerable, however, under‐prioritized mental health burden. Little is known about the mental health burden and services status in the prisons of the region.

**Objectives:**

We aimed to investigate the individual country‐wise prison mental health status in South‐East Asian region.

**Methods:**

We performed a narrative review based on the evidence available in PubMed, Scopus, PsycINFO, Google, and Google Scholar considering the review objectives. We highlighted country perspectives on total population, prison numbers, prisoner numbers, the prevalence of psychiatric disorders and suicide among prisoners, prison mental health services, current challenges, and ways ahead.

**Results:**

We discussed the prison mental health of five countries (Bangladesh, India, Indonesia, Nepal, and Sri Lanka). We found overcrowding (131.4%–215.6%) in the prisons, a high prevalence of psychiatric disorders in the prisons (40%–100%), negligible prison mental health services, and a lack of data on prison suicide with some variations among the five countries. Among the countries, Bangladesh has the highest prevalence (66.4%–100%) of psychiatric morbidity with an absence of a mental health system.

**Conclusions:**

Prison mental health in tSouth‐East Asia is a neglected domain and warrants attention regarding ensuring adequate mental health services to the prisoners as there are high unmet mental health needs and an absence of poorly supported mental health needs.

## Graphical abstract

Prison mental health in South‐East Asia is a neglected domain. There is overcrowding (131.4‐215.6%) in the prisons, a high prevalence of psychiatric disorders in the jails (40‐100%), negligible prison mental health services, and a lack of data on prison suicide with some variations among the five countries.

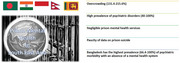

## 1 INTRODUCTION

There are 11 countries (Bangladesh, Bhutan, Democratic People's Republic of Korea, India, Indonesia, Maldives, Myanmar, Nepal, Sri Lanka, Thailand, and Timor‐Leste) within the low‐ and middle‐income country (LMIC) bracket in the South‐East Asia region of World Health Organization (WHO) harboring about one quarter (about 2 billion population) of the global population (Arafat et al., 2024; WHO, 2024). The region has been facing th double burden of diseases with substantial loads of both communicable and noncommunicable diseases. Additionally, there are enduring conflicts (war and politics), natural disasters and calamities, and poverty. Available evidence suggests that 13.2% of the population of the region has mental disorders suggesting about 260 million people with psychiatric illnesses live in the area (WHO, 2023). It is also a suicide‐dense area of the world having about 29% of total suicides with a higher rate than the global rate (10.2 vs. 9 per 100,000, WHO, 2021a). The countries are struggling with the low income of individuals and budgetary allocation to mental health, poor access to mental health care, high out‐of‐pocket expenses, high stigma, and extremely high treatment gap (Arafat & Kar, 2024). Likewise, the state of mental health, forensic psychiatry, and prison mental health has been a neglected area in the region, which is an important problem revealed from studies in the other parts of the world. A recent umbrella review has included 17 meta‐analyses published between 2002 and 2023 that discuss about incidences and prevalence of mental health conditions in the prison population (Favril et al., 2024). Among the prison inmates (irrespective of gender), the 6‐month prevalence of depression, post‐traumatic stress disorder (PTSD), and psychotic disorders was 11.4%, 9.8%, and 3.7%, respectively. Similarly, at the time of arrival to prison, nearly 1/4th of the prisoners qualify for alcohol use disorder and about 2/5th met the diagnosis of drug use disorder (Favril et al., 2024). People in prison having non‐affective psychosis and depression are likely to have a higher possibility of having comorbid substance use disorder (Baranyi et al., 2022). Several recommendations have been given international experts to expand the horizon of interventions for dealing the mental health issues among the prisoners (Murray et al., 2024). However, little is known about the psychiatric morbidity and mental health services status in the prisons in the region as well as in a specific country. Therefore, we aimed to investigate the current status of prison mental health in South‐East Asia focusing on the status of incarceration, psychiatric morbidity among prisoners, and mental health services delivery in an individual country.

## 2 METHODS

### 2.1 Type of review

We performed this narrative, explorative, and unstructured review on the basis of already available sources considering the review objectives.

### 2.2 Selection of studies

We performed a narrative, explorative, and unstructured search to identify the available evidence in PubMed, Scopus, PsycINFO, Google, and Google Scholar. We used “Prison Mental Health in South‐East Asia,” and different combinations mentioning the individual country names (mental health, psychiatric disorders, mental illnesses, prison, prisoners along with the country names—Bangladesh, India, Indonesia, Nepal, and Sri Lanka). We included papers available in the databases and search engines from conception to the search date (January, 2024) discussing the outcome variables considering the study objectives. We also included gray literature and reports from different sources on prison mental health of the selected countries aligned with outcome variables and review objectives.

### 2.3 Selection of countries

We tried to collaborate with local authors of the 11 countries to write their own country perspectives considering the review objectives so that we can mention the local context as much as possible along with the available evidence. We were able to collaborate with five country‐specific authors, that is, Bangladesh, India, Indonesia, Nepal, and Sri Lanka. Therefore, we did not discuss the country perspectives of Bhutan, the Democratic People's Republic of Korea, Maldives, Myanmar, Thailand, and Timor‐Leste.

### 2.4 Outcome variables

We highlighted country perspectives on total population, prison numbers, prisoner numbers, occupancy rate, rate of psychiatric disorders, perspectives on suicidal and self‐harm behavior among prisoners, prison mental health services status, individual country specific current challenges, and ways ahead. In this review, we used “psychiatric disorder,” “psychiatric morbidity,” “mental illness,” and “mental disorder” interchangeably.

### 2.5 Ethical permission

As we reviewed the already available sources, we did not seek for a formal ethical clearance.

## 3 RESULTS

### 3.1 Bangladesh

#### 3.1.1 Population and legal structure

The recent (2022) *Population & Housing Census* of Bangladesh found about 170 million populations in Bangladesh (Bangladesh Bureau of Statistics, 2023). The legal and judicial systems mostly evolved from the British administration (Ahmed et al., 2024). Several laws guide the prison mental health in Bangladesh like the Ministry of Law, Justice and Parliamentary Affairs (2018), the Code of Criminal Procedure (1898), the Bangladesh Jail Code (2006 revised edition), the Penal Code (1860), and the Police Ordinance (2008 draft) (Ahmed et al., 2024; Ashraf et al., 2022). The Mental Health Act promotes the rights of persons with psychiatric illness and advocates the need for mental health services for prisoners (Ministry of Law, Justice and Parliamentary Affairs, 2018).

#### 3.1.2 Prison and prisoners

Bangladesh has 68 prisons (13 central and 55 district jails) with a capacity of 42,866 prisoners (World Prison Brief, 2024a). There is one female prison near Dhaka and the rest 67 have both male and female sections (Khan et al., 2021). There were 84,851 prisoners in December 2023 (9 December) with an incarceration rate of 50 per 100,000 population (World Prison Brief, 2024a) and an occupancy rate is about 200%. About two‐thirds of the prisoners were at the pretrial stage and about 4% were females (World Prison Brief, 2024a).

#### 3.1.3 Prison mental health services

There were 141 posts for doctors in the 68 prisons and among them, 137 were found vacant (Islam, 2022). Although every prison has an allocation for mental health professionals, none of them was available in any prison in the country (Islam, 2022). There are 15 beds for hospital admission under forensic psychiatry in Bangladesh at the *National Institute of Mental Health (NIMH)*, Dhaka (Ahmed et al., 2024). There are no official prison mental health services and no screening strategy for assessing mental disorders is available in Bangladesh to date (Khan et al., 2021). Mental health experts or psychiatrists do not attend prisons for any clinical services. However, some activities from nongovernment organization (Dhaka Ahsania Mission) have been identified focusing the substance‐related issues and training the staff for stress management, anger control, and mindfulness (Khan et al., 2021). Prisoners with mental illness (suspected) are usually sent to nearby hospitals with mental health services accompanied by two police officers. At the department of forensic psychiatry of NIMH, both inpatient and outpatient services are provided free of cost for the prisoners. Some psychotropics, such as conventional antipsychotics, mood stabilizers (lithium and valproate), sedative‐hypnotics, and amitriptyline, are available free of cost.

#### 3.1.4 Prevalence of psychiatric disorders among prisoners

Several studies have been conducted among the prison population in Bangladesh even though studies are scattered and conducted among the referred prisoners (Ahmed et al., 2024). We found the first study was done at Pabna Mental Hospital among 56 prisoners referred from Pabna Jail (Chowdhury et al., 1998). All the prisoners had some sort of psychiatric disorder and 91% of them had the illness before committing the crime (Chowdhury et al., 1998). Another study assessed 67 male prison inmates referred by Dhaka Central Jail authority and found that 91% had mental disorders (Mullick et al., 1998). Both studies were conducted among prisoners referred to as out‐patients (Chowdhury et al., 1998; Mullick et al., 1998). One study conducted by Hamid et al. (2005) assessed 48 inpatient‐admitted prisoners and found the prevalence of mental disorders was 85.4%. One recent study conducted by Yasmin et al. (2022) among 127 referred patients from the prison and found the prevalence of mental illness was 94.4%. Both studies found psychotic disorder as the major morbidity. We found a single study assessing only female prisoners that assessed 250 female prison inmates by structured diagnostic instrument. The study found the point prevalence of psychiatric disorders was 66.4% and epression was the major morbidity (depression 30.4%) (Hasan et al., 2004). We did not find any country‐wide prison‐based study that has recommended establishing regular screening for psychiatric illness in the country (Khan et al., 2021). It is prudential to consider that in Bangladesh, there is high stigma and extreme lack of mental health support in the prison settings; usually, patients with major symptoms are referred to mental health care services providers, which may be an important attribution for the high rate of psychiatric as well as psychotic disorders.

#### 3.1.5 Suicide in prison

We found news reports mentioning suicides among prisoners. One report mentioned that 12 suicides were noted between January 2020 and September 2033 (Rashid, 2023). However, we did not find any publicly available data on suicidality among prisoners in Bangladesh.

#### 3.1.6 Challenges and ways ahead

The primary challenge of prison mental health in Bangladesh is a lack of cognigence regarding the importance of prison mental health. There are lack of collaboration and training among mental health professionals and legal authorities. In addition, Bangladesh needs intense efforts as there is no formal prison mental health in the country.

### 3.2 India

#### 3.2.1 Prison and prisoners

The National Crime Records Bureau (NCRB) releases the statistics of Indian prisons every year (NCRB, 2023). The latest report of “Prison statistics of India” released in December 2023 mentions that by the end of December 2022, there are 1330 prisons in India, with a total capacity of 436,266, and an occupancy of 573,220, which amounts to an occupancy of 131.4% (Institute for Crime & Justice Policy Research, 2023; NCRB, 2023). Ninety‐seven out of the total prisoners are transgender, whereas 23,772 are females and the rest are males. Out of the total prisons in India, 34 are dedicated to women and 10 are borstal schools (NCRB, 2023). The highest number of prisoners are there in the state of Uttar Pradesh (15.5% of total prisoners in India) followed by Bihar (10.9% of total prisoners in India) and Madhya Pradesh (6.8% of total prisoners in India). The number of foreign prisoners in India is 6283 (1.1% of the total prisoners).

#### 3.2.2 Prison mental health services

The India Justice Report 2022, which analyzed the Prison Statistics of India 2021, mentions that the correction and rehabilitation facility of the Indian prisons is grossly inadequate and poor prison conditions result in significant psychological distress for the prison inmates (India Justice Report, 2023). To address the mental health issues of prison inmates, there are only 68 sanctioned posts of psychiatrists or psychologists, but there are only 33 psychiatrists/psychologists exist, which corresponds to one mental health professional per 16,789 prison inmates (India Justice Report, 2023). There has been a significant increase in unnatural deaths (particularly due to suicide, murder by prison inmates, and negligence) in recent years (India Justice Report, 2023).

#### 3.2.3 Prevalence of psychiatric disorders among prisoners

Mental health issues are common among the prisoners. A recent systematic review that estimated the burden of noncommunicable diseases among Indian prisoners found that psychiatric disorders like—depression, suicidal ideation, and substance use are common (Manna et al., 2022). A study in a central jail in Odisha (India) found that more than 50% of the jail inmates have moderate to severe depression (Tripathy et al., 2022). Being accused of a crime, having less psychological support, and having a higher education level are the variables that are strongly linked to depression in prison inmates as found in the above study (Tripathy et al., 2022). A retrospective study by Gowda et al. (2019) evaluated female forensic hospitalized patients in Karnataka and found that schizophrenia, mood disorders, and anxiety disorder are the common mental health issues, and these conditions significantly affect the life of female prisoners (Gowda et al., 2019). A study from Kerala revealed that more than two third of prisoners suffer from a mental illness currently, of which substance use disorder is the most common psychiatric diagnosis. Suicide risk of moderate to high severity has been reported in about 4% of the prisoners (Ayirolimeethal et al., 2014). A lots of variations seen in the prevalence of different mental health issues among prison inmates residing in various corners of India, as reported in several studies (Goyal et al., 2011; Kumar & Daria, 2013; Pallavi et al., 2014).

#### 3.2.4 Suicide in prison

Suicide is another major concern in Indian prisons. Over the past two decades, the suicide rate in Indian prisons has almost doubled (Gowda et al., 2021). Hanging is the common mode of suicide in Indian prisons (Gowda et al., 2021; NCRB, 2023). Substance use disorder is a prominent mental health issue reported among the prisoners. In a qualitative study among prison inmates, it was found that some important factors like—coping‐related issues, exposure to substances, interpersonal issues in the prisoners, and conflicts are closely associated with substance use (Ghosh et al., 2023). Vajawat et al. (2023) reported that prisoners with mental health issues, who are hospitalized, often have severe mental illnesses like schizophrenia and related psychotic disorders, alcohol use disorders, and mood disorders. If these patients are provided with adequate mental health care and support, the outcome is often quite satisfactory (Vajawat et al., 2023).

#### 3.2.5 Challenges

To deal with mental health issues and substance use, the identification of these conditions is important. The paucity of mental health manpower stands as a major obstacle in the early identification of the mental health issues of prisoners in India. A recent Indian study attempted to test the feasibility of a peer support program for prisoners in an Indian prison setting (Thekkumkara et al., 2023). It was found that this is a feasible and effective model of screening for common mental disorders, substance use, and suicidality among the incarcerated population (Thekkumkara et al., 2023). Thekkumkara et al. (2022) developed and validated the peer support program for the incarcerated population with mental health issues, including substance use disorder. Sureka et al. (2014) did a parallel randomized controlled trial lasting 6 months using Sudarshan Kriya Yoga practices and found that there is a significant improvement in general health, self‐control, positive well‐being, vitality, and reduction in anxiety and depression in the prison inmates. Yoga and meditation practices can be used as a positive mental well‐being intervention to reduce the risk of mental illness in prisoners. As it is an intervention that can be delivered in groups and peer training may be useful in meeting the manpower constraints.

#### 3.2.6 Ways ahead

The paucity of mental health human resources at the prisons significantly compromises the mental health care delivery and to bridge the gap teleconsultation facilities may be a feasible option. A tertiary care facility in South India successfully attempted to provide mental health care facility to prisoners suffering from schizophrenia, mood disorders, substance use disorders, anxiety disorders, and epilepsy (Agarwal et al., 2019). It can be replicated in other parts of the country to bridge the treatment gap for mental illnesses among prisoners. The major challenges in the Indian prison setting continue to be overcrowding, unhealthy and hostile atmosphere, poor health care facilities, and risk of high‐risk behavior including sexual contact leading to increasing vulnerability for sexually transmitted diseases including HIV and AIDS (Bhaumik & Mathew, 2015). There is a need to address the challenges at the prison that increase vulnerability to mental illnesses.

### 3.3 Indonesia

#### 3.3.1 Prison and prisoners

Indonesia is the world's fourth most populous country, following India, China, and the United States. As of mid‐2023, its population reached 278.69 million, as the Central Statistics Agency reported in 2024. Concurrently, the number of incarcerated individuals is on the rise. By 2023, Indonesia boasted 301 prisons, 161 jails, and 33 prisons for children, locally called the Special Children's Development Institutes. Although designed to accommodate up to 128,656 individuals, these facilities currently house 228,204 occupants, indicating a 177% overcapacity issue (Indonesian Ministry of Law and Human Rights, 2024).

#### 3.3.2 Prevalence of psychiatric disorders among prisoners

The health issues of Indonesian prisoners have become a focal point for authorities. Several concerns have emerged regarding health within prisons, such as insufficient nutrition intake, with over half of inmates experiencing deficiencies and most having poor nutritional status (Dewi et al., 2017). Additionally, there is a heightened risk of tuberculosis infection (Kurniawan et al., 2024; Thuffi & Herdayati, 2018) and mental health challenges.

Psychological distress is prevalent among prisoners due to the harsh prison environment, overcrowding, and limited access to mental health services. It was found that more than half of women prisoners in Jakarta had signs of depression, and it was associated with age and recidivism status (Juliane & Machmud, 2020). More than half of female prisoners were also found to suffer from psychotic symptoms, and it was not associated with the duration of imprisonment (Adhyatmac et al., 2015). However, another study found that more than half of prisoners had good psychological well‐being (Hidayati et al., 2021). Given the prevalence of psychological distress among prisoners, it is imperative to prioritize mental health support services tailored to address varying needs across different prison settings and demographics.

Between December 2010 and November 2012, a comprehensive cross‐sectional investigation encompassed 1808 inmates across five prisons on Java Island. Employing the *Mini International Neuropsychiatric Interview*, the study aimed to identify various mental health conditions among the incarcerated population. Results indicated that over half (65%) of the prisoners exhibited symptoms indicative of mental disorders. Further examination revealed that 5.5% had psychotic disorders, 20.5% experienced mood disorders, 32.6% were afflicted with substance use disorders, and 41.5% encountered neurotic issues (Mardiati & Anindyajati, 2013).

#### 3.3.3 Prison mental health services

Despite being relatively low, the inmates utilize the counseling service whenever available. The problems they consulted during the counseling include challenges within the family (59%), issues with partners (20%), difficulties in the prison environment (6%), apprehension about release (6%), struggles with self‐esteem (6%), and employment concerns (Subandi et al., 2022). To address the diverse range of concerns expressed by inmates during counseling sessions, it is recommended to implement targeted interventions and support programs tailored to meet their specific needs and improve overall well‐being.

#### 3.3.4 Ways ahead

The epidemiological study on prisoner mental health in Indonesia is crucial to be conducted. The provided data are relatively outdated and only conducted on the island of Java, leaving other settings across Indonesia unknown. Screening the prisoner's mental health is significant to understanding their mental health status, which is essential for planning and intervention strategies. It also helps identify areas of concern and prioritize resources for mental health services within the prison system, reduce recidivism rates, and promote successful reintegration into society.

### 3.4 Nepal

#### 3.4.1 Prison and prisoners

Department of Prison Management under the Ministry of Home Affairs is responsible for overseeing the management of prisons in Nepal. There are 74 prisons in Nepal, with 27,550 prisons, which is 90 per 100,000 population of Nepal. Among them, 5.4% are women and 3.3% are juveniles/minors/young prisoners. The occupancy rate is 153.7% of official capacity (World Prison Brief, 2024b; Government of Nepal, 2024).

#### 3.4.2 Legal structure

Nepal's Prison Act was formulated in 1963 followed by many amendments. Regarding mental health, it states “(1) Treatment of the physically or mentally sick detainees or prisoners shall be done by the government doctor. Provided that, if any detainee or prisoner wishes to have his/her treatment done by any other doctor at his/her own cost, permission shall be granted to have such treatment as prescribed” (Nepal Law Commission, 2024).

#### 3.4.3 Prevalence of psychiatric disorders among prisoners

A study from a prison in Eastern Nepal among 434 randomly selected prisoners using the *Center for Epidemiologic Studies Depression* scale and semi‐structured questionnaire showed that the prevalence of depression was 35.3% and 2.3% reported suicidal ideation during imprisonment and 0.9% had suicide attempts inside the prison. Depression was significantly associated with previous incarceration and frequent appointments when encountering health problems (Shrestha et al., 2017).

A cross‐sectional study among 92 respondents from female‐only prison in Nepal using the *Beck Depression Inventory* showed that 37 (40.1%) had symptoms of depression. No association was found between the level of depression and sociodemographic characteristics, imprisonment characteristics, and substance use characteristics of the prisoners. Regarding knowledge, eight (8.7%) had knowledge about their right to mental health (Regmi et al., 2019).

Another cross‐sectional done at Nakkhu Jail in Kathmandu among 490 prisoners using Modified Mini Screen found that the psychiatric morbidity was positive among 10.4% of prisoners. Suicidality was present among 3.5%, and 3.1% had PTSD. Psychiatric morbidity was associated with length of stay in prison and satisfaction with living facilities (Parajuli, 2023).

A report published by the *Office of the High Commissioner for Human Rights in Nepal* in 2008 reported that in Dhulikhel prison, 84% of the mentally disabled detainees are detained in relation to homicide. The prison provides a separate section for severely mentally disabled detainees and this section represents the poorest living conditions in the prison (Office of United Nations High Commissioner for Human Rights in Nepal, 2008).

#### 3.4.4 Prison mental health services

The Central Jail Hospital has a dedicated post for a psychiatrist. There is a hospital run in the prison at Nakkhu, which has been proposed to develop into a prison mental hospital. There have been periodic mental health assessments and treatment of inmates of prison where the psychiatrists, psychologists, and nongovernment organizations have been proving mental health care. In addition, mental health assessment and support for children detained for criminal offense has been initiated (WHO, 2021a). However, these are bits and pieces that would not be enough to cater the mental health needs of the prison population. There is a definite need for psychiatric and substance abuse care in correctional settings to improve the health status of the prison population of Nepal.

### 3.5 Sri Lanka

#### 3.5.1 Population and legal structure

The population of Sri Lanka is about 22 million (United Nations Population Fund, 2024). The regulation of mental health within Sri Lankan prisons is governed by various legal frameworks, including the Mental Disease Ordinance (Last amended as Act 27 of 1956), the Prisons Ordinance (No. 16 of 1877), Code of Criminal Procedure Act (No. 15 of 1979), Penal Code (Ordinance No. 2 of 1883), and the Police Ordinance (No 16 of 1865). Furthermore, there is anticipation regarding an upcoming mental health act, expected to prioritize addressing the requirements of mentally ill prisoners.

#### 3.5.2 Prison and prisoners

Sri Lanka's prison system comprises four closed prisons for convicted individuals, 18 remand prisons, 10 work camps, 2 open prison camps, 1 training school, 2 correctional centers for youthful offenders, and 23 lockups. Notably, drug rehabilitation programs are conducted in two work camps, one open prison, and one correctional center specifically designed for youthful offenders (Statistics Division of Prison Head Quarters, 2023).

Statistically, the daily average of convicted prisoners stands at 8416, alongside 13,563 un‐convicted or remanded prisoners. In the year 2022, there were 108,250 un‐convicted admissions, with 104,157 being male and 4093 females. Among these, 161 individuals were under the age of 16, whereas 7613 fell between the ages of 16 and 22 (Statistics Division of Prison Head Quarters, 2023).

Furthermore, in 2022, a total of 30,331 prisoners were convicted, comprising 29,941 males and 390 females. Among these, 2304 were under the age of 22. Notably, nearly two‐thirds of these convictions (19,094) were related to narcotic drug offenses (Statistics Division of Prison Head Quarters, 2023). However, it is essential to recognize that not all of these individuals can be assumed to be drug users, highlighting the imperative for specialized considerations in rehabilitation and addressing mental health needs. Additionally, 47 individuals received death sentences, 21 received life sentences, and 3 were categorized as youthful offenders, with the remaining 11,166 convicted for other offenses (Statistics Division of Prison Head Quarters, 2023). It should be acknowledged that the death penalty has not been implemented in Sri Lanka since 1976 (Human Right Commission Sri Lanka, 2020).

In November 2020, the Human Rights Commission of Sri Lanka released a comprehensive report on prison conditions following a thorough 2‐year study involving interviews with relevant stakeholders. The findings indicated that prisoners in Sri Lanka endure substandard living conditions and lack access to essential services such as health care and rehabilitation, primarily due to severe overcrowding and staffing shortages. Certain vulnerable groups, including death row inmates, women, young offenders, foreign nationals, individuals detained under anti‐terrorism laws, and prisoners with disabilities, face additional challenges. The report strongly advocated for policymakers to address these specific issues to ensure equitable access to opportunities for reform among all prisoners (Human Right Commission Sri Lanka, 2020).

#### 3.5.3 Psychiatric morbidity among prisoners

The available literature on the mental health of incarcerated people is still limited in scope. A descriptive cross‐sectional study conducted among inmates of a main prison in Southern Sri Lanka revealed notable findings. Among 845 participants, 17.5% reported psychiatric disorders, whereas 3.9% reported experiencing trauma. Additionally, 31.3% reported psychological stress, highlighting the prevalence of mental health concerns among this demographic. The study identified a substantial history of substance use among inmates. The study reported past usage rates of 66.2% for smoking, 62.1% for alcohol, 12.6% for heroin, and 16.9% for cannabis, underscoring the prevalence of substance abuse within the prison population (Wickramatilake, 2023).

Similarly, a cross‐sectional study conducted at Welikada prison, the largest correctional facility in Sri Lanka, unveiled alarming statistics regarding substance use disorders among inmates. Lifetime substance use disorder was observed in 75.6% of participants, with 24.4% meeting the criteria for current substance use disorder. Moreover, high prevalence rates were noted for alcohol (56.8%) and tobacco (67%) use disorders, along with significant percentages for cannabis (42%) and opioid (25.6%) dependence (Hapangama et al., 2021).

#### 3.5.4 Suicidal and self‐harm behavior among prisoners

Few available studies focus primarily on suicidal and self‐harm behavior among prisoners, with a lesser degree on drug use and mental illness. An institutional‐based cross‐sectional analytical study involving 1730 participants revealed a prevalence of 22.7% for suicidal behavior among prison inmates. Factors associated with such behaviors included psychological distress, younger age, female gender, legal circumstances, familial issues, and exposure to violence and trauma (Suranga & Vidanapathirana). Another study was conducted in juvenile detention centers to examine self‐harm among 181 young individuals. Findings showed 43% had a history of self‐harm, with 25% indicating suicidal intent. Notably, 65% self‐harmed impulsively. Factors linked to self‐harm included gender, past sexual abuse, peer influence, and prior self‐harm thoughts. High rates of substance use, bullying, parental incarceration, and exposure to suicide were also noted. The study recommends strategies to identify and prevent self‐harm, including tailored interventions focusing on impulsivity, to improve outcomes for this vulnerable group (Hettiarachchi et al., 2018). According to a report by the Human Rights Commission of Sri Lanka, a considerable percentage of prisoners, particularly those detained under the Prevention of Terrorism Act, have reported attempted self‐harm and suicide while incarcerated (Human Right Commission Sri Lanka, 2020).

#### 3.5.5 Mental health services in prison

Currently, Sri Lanka is equipped with both Forensic Psychiatry and Correctional Psychiatry services. The nation hosts three Forensic Psychiatry units, with the largest, oldest, and most established unit situated at the NIMH, Sri Lanka. This facility operates as a medium‐secure unit with a capacity of 120 beds for both male and female individuals. These beds primarily serve for the evaluation and treatment of offenders referred from courts for forensic psychiatry assessments. The remaining two units, established more recently, are located at the Teaching Hospital Karapitiya in the Southern Province of Sri Lanka (established in 2017) and the National Hospital Kandy in the Central Province of Sri Lanka (established in 2019). Notably, these units lack dedicated inward facilities, leading to the admission of forensic assessment and treatment cases into General Psychiatry wards.

General Psychiatry services, in areas devoid of specialized forensic psychiatry units, also receive court referrals for forensic psychiatry evaluations. During this interim period until preliminary assessments are concluded, individuals may be admitted to General Psychiatry units for a brief duration, despite the absence of specialized forensic assessment teams. Apart from individuals referred to for forensic psychiatry evaluations by courts, those deemed not guilty by reason of unsoundness of mind or unfit to plead are admitted to the forensic unit of the NIMH in accordance with the criminal procedure code of Sri Lanka (Code of Criminal Procedure Act, No. 15 of 1979).

Correctional psychiatric services are coordinated among prison mental health services, forensic psychiatry units, and general psychiatry units. The latter two operate under the *Ministry of Health*, whereas prison health services fall under the purview of two separate ministries.

Prison Medical officers, including prison medical officers of mental health, are hired by the Ministry of Health, which is in charge of obtaining drugs and medical equipment. Nurses and dispensers for prison hospitals are recruited by the Department of Prisoners, which oversees these facilities. According to prison statistics 2023, the entire prison system employed 46 medical officers, 23 registered medical practitioners, and 35 nurses (Statistics Division of Prison Head Quarters, 2023). Prior to 2020, only five prisons had established regular psychiatric clinics over the preceding 2 years, with a limited provision of correctional psychiatry care through visits by forensic psychiatrists to one prison hospital, as noted in the *Human Rights Commission* report (Human Right Commission Sri Lanka, 2020).

Furthermore, on the basis of the author's experiential knowledge and understanding of current services, it is observed that only two prisons have dedicated medical officers for mental health. Furthermore, two prisons are visited by General Psychiatry teams that provide in‐prison mental health treatments, and three other prisons are visited by forensic psychiatry units.

Prisons bring prisoners to outpatient psychiatry clinics when medical officers deem specialized attention necessary or when mental health services are unavailable within the respective prison. However, accessing external services is limited due to human resource scarcity within the prison system. Whenever inward treatment is necessary for prisoners (both remandees and convicted individuals), they are admitted either to the nearest General Psychiatry ward or to a forensic psychiatry unit at the NIMH. Both the mental disease ordinance and the prison ordinance have provisions for NIMH admissions (Mental Disease Ordinance, Last amended as Act 27 of 1956, The Prisons Ordinance, No. 16 of 1877*)*.

#### 3.5.6 Challenges

As highlighted in the Human Rights Commission report, the absence of seamless coordination between the Ministry of Health and the Department of Prisoners presents a glaring challenge within the Sri Lankan prison health care system. This lack of synergy, compounded by overlapping responsibilities, serves as a formidable barrier to the effective administration and delivery of health care services to incarcerated individuals (Human Right Commission Sri Lanka, 2020).

#### 3.5.7 Ways ahead

These findings underscore the urgent need for structured screening programs and improved mental health facilities within prison institutions. Implementation of mandatory mental health modules focusing on coping strategies for psychological distress and mental health promotion among all inmates is imperative. Additionally, interventions addressing substance abuse and trauma‐related issues are crucial for enhancing the well‐being of incarcerated individuals. To address this issue, a unified approach is imperative, whereby the provision of health care to prisoners is consolidated under a single authority. Such a restructuring would allow for the establishment of dedicated, well‐trained multidisciplinary teams, attuned to the unique needs and complexities inherent within correctional health care settings. By identifying and responding to these needs with tailored and comprehensive interventions, the system can strive toward delivering equitable and high‐quality health care services to prisoners across the board.

## 4 DISCUSSION

### 4.1 Major findings of the review

The review revealed overcrowding in the prisons of the five SEAR countries; highest in Sri Lanka (215.6%), Bangladesh (200%), followed by Indonesia (177%), Nepal (153.7%), and India (131.4%) (Table 1). The highest proportion of female prisoners was noted in Nepal (5.4%), followed by Sri Lanka (4.4%), India, and Bangladesh (about 4%). Among the countries, Bangladesh has no formal mental health system, no publicly available data on prison suicide and the highest prevalence of psychiatric disorders (66.4%–100%). One study in Bangladesh found the prevalence 100% among 56 patients referred from 56 from Pabna Jail (Chowdhury et al., 1998). It is explainable considering that due to extreme lack of awareness and mental health services in prison setting only patients with severe symptoms were referred to for mental health care. It was the first published study on prisoners’ mental health in the country about 25 years ago, whereas diagnoses were assigned by clinical assessment at out‐door setting. The psychiatric morbidity was about 50% in India, 65% in Indonesia, and about 40% in Nepal. Mental health services are scarce in comparison to demand in the other four countries (India, Indonesia, Nepal, and Sri Lanka). Prison suicide data are available in India, which have been increasing; suicidal ideation was assessed in Nepal; suicidal behavior and self‐harm were measured in Sri Lanka.

**TABLE 1:** Prison mental health status is South East Asia (World Prison Brief, 2024c).

Variable	Bangladesh	India	Indonesia	Nepal	Sri Lanka
Occupancy (%)	About 200	131.4	177	153.7	215.6 (August 2023)
Female (%)	About 4	4.1	4.7	5.4	4.4
Any psychiatric disorder (%)	66.4–100	About 50	65	About 40	Limited study; 17.5–24.4
Prison mental health services	No	Available and scarce	Counseling and scarce	Available and scarce	Available and scarce
Prison suicide data	Not available	Available	Limited access to data available or not publicly available	Limited data available	Limited data available

All the SEA countries belong to the LMIC group. Prisoner's health issues, particularly those involving mental health, are matters of significant concern in LMICs (Baranyi et al., 2019). In the SEAR, the common disabling mental health conditions are—depressive disorder, anxiety disorder, and schizophrenia (WHO, 2023). Although there are an inadequate number of studies along with some issues with study methods, there is a high prevalence of psychiatric disorders in SAER countries. Similar findings are noted in other studies also (Gómez‐Figueroa & Camino‐Proaño, 2022). Among the prisoners, mental health conditions are more prevalent than the general population. A recent systematic review and meta‐analysis that involved more than 14,000 prison inmates from 13 LMICs found that the prevalence of psychosis, depression, alcohol use disorder, and other drug use disorder to be 6.2%, 16.0%, 3.8%, and 5.1%, respectively (Baranyi et al., 2019).

The scarcity of mental health services is alarming in the region due to its overall scarcity of human resources, allocation to mental health, and the overlaps between the Home and Health ministries (Arafat & Kar, 2024). Additionally, the enduring status of human rights in the countries along with the political environment could affect the services (Amnesty International, 2024). Similar conditions of nonexistence plans and polices for mentally ill offenders were noted in South Africa (Sukeri et al., 2016). However, equal mental health care to the community people for prisoners has been attempted in the United Kingdom and Europe (Forrester et al., 2013) shifting the view from prisoner to patient concept (Birmingham, 2003). It is important to follow international standards of prison mental health services and ensure screening in several stages and endure different prevention strategies (Forrester et al., 2018; Senior et al., 2012).

### 4.2 Implications of study results

The study indicates improper attention to the mental health of the prisoners in SEAR, a densely populated area with an LMIC background. Immediate attention is warranted from countries, regional bodies, and international organizations to improve the status quo. There should be a separate prison mental health services system in every country. The initiation of tele‐psychiatry could be an important step in this region as it has already been tested in India (Agarwal et al., 2019). International humanitarian organizations could engage more actively in the countries with supports for mental health. During the post‐COVID times, tele‐psychiatric consultation services are widely being adopted. The development of teleconsultation units in jail hospitals may help in integrating general health facilities with mental health care facilities and may help in minimizing the treatment gap (Karachaliou et al., 2023).

### 4.3 Strength and limitations of the review

This is the first comprehensive review focusing on the psychiatric morbidity among prisoners and the mental health services system in the prisons in South‐East Asia. There are several important limitations of this review. This review focused on readily available evidence and there is a dearth of studies on prison mental health in South‐East Asian countries. Therefore, this review may not reflect the actual scenario. Second, a systematic search was not used to identify the existing literature that may exclude some articles. Third, there was a lack of available data regarding suicide in prison. Fourth, we used “psychiatric disorder,” “psychiatric morbidity,” “mental illness,” and “mental disorder” interchangeably, which may not be the same. Fifth, there is heterogeneity in assigning psychiatric diagnoses. We considered any psychiatric disorder mentioned in the available articles. It is important to consider that different studies in different settings used different methods and tools to assign mental disorders. Sixth, we discussed the contexts of 5 out of 11 countries. Inclusion of the perspectives from all the 11 countries would reflect the comparison better.

## 5 CONCLUSION

Prison mental health in South‐East Asia is a neglected domain and warrants attention regarding ensuring adequate mental health services to the prisoners as there are high unmet mental health needs and an absence to poorly supported mental health services. Prisoners have high psychiatric morbidity than the community people with low or negligible mental health services in SEAR. Policymakers and different stakeholders including human rights organizations should consider the mental health of the secluded section of society.

## AUTHOR CONTRIBUTIONS

**S. M. Yasir Arafat**: Conceptualization; investigation; writing—original draft; writing—review and editing; methodology; supervision; project administration. **Sujita Kumar Kar**: Methodology; writing—original draft; writing—review and editing. **Chittahari Abhayanayake**: Writing—original draft. **Pawan Sharma**: Writing—original draft. **M. Marthoenis**: Writing—original draft.

## CONFLICT OF INTEREST STATEMENT

The authors declare that there are no conflicts of interest.

## FUNDING INFORMATION

We did not receive any funding for this study.

### PEER REVIEW

The peer review history for this article is available at https://publons.com/publon/10.1002/brb3.70004

## Data Availability

No data were generated for this study.
